# Individual Brain Morphological Connectome Indicator Based on Jensen–Shannon Divergence Similarity Estimation for Autism Spectrum Disorder Identification

**DOI:** 10.3389/fnins.2022.952067

**Published:** 2022-06-28

**Authors:** Ting Yi, Weian Wei, Di Ma, Yali Wu, Qifang Cai, Ke Jin, Xin Gao

**Affiliations:** ^1^Department of Radiology, Hunan Children’s Hospital, Changsha, China; ^2^College of Information Science and Technology, Nanjing Forestry University, Nanjing, China; ^3^Department of Child Health Care Centre, Hunan Children’s Hospital, Changsha, China; ^4^Shanghai Universal Medical Imaging Diagnostic Center, Shanghai, China

**Keywords:** identification, global metric, nodal metric, autism spectrum disorder, individual brain morphological connectome

## Abstract

**Background:**

Structural magnetic resonance imaging (sMRI) reveals abnormalities in patients with autism spectrum syndrome (ASD). Previous connectome studies of ASD have failed to identify the individual neuroanatomical details in preschool-age individuals. This paper aims to establish an individual morphological connectome method to characterize the connectivity patterns and topological alterations of the individual-level brain connectome and their diagnostic value in patients with ASD.

**Methods:**

Brain sMRI data from 24 patients with ASD and 17 normal controls (NCs) were collected; participants in both groups were aged 24–47 months. By using the Jensen–Shannon Divergence Similarity Estimation (JSSE) method, all participants’s morphological brain network were ascertained. Student’s *t*-tests were used to extract the most significant features in morphological connection values, global graph measurement, and node graph measurement.

**Results:**

The results of global metrics’ analysis showed no statistical significance in the difference between two groups. Brain regions with meaningful properties for consensus connections and nodal metric features are mostly distributed in are predominantly distributed in the basal ganglia, thalamus, and cortical regions spanning the frontal, temporal, and parietal lobes. Consensus connectivity results showed an increase in most of the consensus connections in the frontal, parietal, and thalamic regions of patients with ASD, while there was a decrease in consensus connectivity in the occipital, prefrontal lobe, temporal lobe, and pale regions. The model that combined morphological connectivity, global metrics, and node metric features had optimal performance in identifying patients with ASD, with an accuracy rate of 94.59%.

**Conclusion:**

The individual brain network indicator based on the JSSE method is an effective indicator for identifying individual-level brain network abnormalities in patients with ASD. The proposed classification method can contribute to the early clinical diagnosis of ASD.

## Introduction

Autism spectrum disorder (ASD) is a heterogeneous neurodevelopmental disorder that manifests in early childhood, with core symptoms of language and social communication disorders, decreased engagement, and repetitive stereotypes of limited activity (Li et al., 2017; Lord et al., 2018). It has become a major global public health problem due to its high incidence and disability rate (Lyall et al., 2017). Usually, such disease can be diagnosed by multidisciplinary professionals (pediatricians, psychiatrists, or psychologists) ***via*** clinical scales, symptoms, and signs. However, this approach is only sensitive enough to identify most children with ASD in whom parents have already noticed symptoms (Mandell and Mandy, 2015). Therefore, it is necessary to explore a reliable indicator to distinguish preschool children with ASD from normal controls (NCs).

Many neuroimaging techniques are widely used to explore pathophysiological changes in the anatomy and function of patients with ASD, such as structural magnetic resonance imaging (sMRI), diffusion tensor imaging (DTI), and blood oxygen level dependent (BOLD), sMRI has attracted attention for its ability to provide multidimensional indicators, such as gray matter (GM) volume, cortical thickness, and gyrification index (Courchesne et al., 2001; Courchesne, 2002; Carper and Courchesne, 2005; Schumann et al., 2010; Ecker et al., 2013; Elsabbagh and Johnson, 2016). Previous sMRI studies have shown that patients with ASD have brain network alterations (Hazlett et al., 2017), abnormal connections (Ecker et al., 2015), and local overconnectivity with specific areas (Lewis et al., 2014), such as the frontal and occipital regions (Rane et al., 2015). It is well known that brain morphological network features detected in patients with ASD could help distinguish these individuals from NCs, and its classification accuracy for ASD ranges from 75.4 to 90.39% (Gao et al., 2020). However, the patients in the studies mentioned above were children over 7 years of age. How brain networks affect specific brain regions in preschool children is still worth exploring further.

As mentioned above, the individual brain morphological networks detected in patients with ASD can help separate these individuals from NCs and reveal relevant pathophysiological mechanisms. Therefore, it is necessary to build a frame of morphological networks for ASD early diagnosis. However, most studies have focused on group-level network methods for morphological network modeling, ignoring information at the individual level (Wang et al., 2016). In this paper, the Jensen–Shannon Divergent Similarity Estimation (JSSE) method (Zhu et al., 2021) was used to construct individual brain networks for preschool children with ASD. Student’s *t*-test was used to select critical features of brain networks between groups. There are two primary aims of this study: (1) To discover altered patterns of individual brain connectome, including morphological connectivity, node graph metrics, and global graph metrics, in preschool children with ASD. (2) To achieve accurate classification of preschool children with ASD and NCs.

## Materials and Methods

### Participants

Only children aged between 2 and 5 years were included in this study. A total of 24 preschool children with ASD (18 male and 6 female, 32.29 ± 7.32 months) who were diagnosed with ASD based on DSM-5, Gesell Developmental Scales (Gesell); Autism Behavior Checklist (ABC); the Modified Checklist for Autism in Toddlers (M-CHAT); Clancy Autism Behavior Scale (CABS), scanned with sMRI, were consecutively enrolled in this study between January 2019 and December 2020. We excluded patients with a history of hypoxic ischemic encephalopathy, head trauma, psychiatric disorders, and substance use disorder. Seventeen typical developmental NC groups, including 5 males and 12 females, aged 34.94 ± 7.86 months, matched for similar ages, and sex distributions were randomly recruited to obtain normative data. Detailed clinical participants’ information can be found in Table 1. None of the NCs had a history of cognitive impairment or neurological or psychiatric disorders. The study was approved by the Ethics Committee of Hunan Children’s Hospital. After signing informed consent, each subject was examined by magnetic resonance imaging (MRI).

**TABLE 1:** Local and global graph metrics of the morphological brain connectome.

Local graph metrics	Global graph metrics
Degree centrality (DC)	Assortativity (*Ar*)
Nodal efficiency (Ne)	Modularity score (*Q*)
Betweenness centrality (BC)	Hierarchy (*Hr*)
Nodal characteristic path length (N*L_p_*)	Global efficiency (*E*_*glocal*_)
Nodal clustering coefficiency (N*C_p_*)	Local efficiency (*E*_*local*_)
Nodal local efficiency (NLe)	Clustering coefficient (*C_p_*)
	Normalized clustering coefficient (γ)
	Normalized characteristic path length (λ)
	Small-world (σ)
	Characteristic path length (*L_p_*)
	Synchronization (*Sr*)

### Data Acquisition

All participants were scanned using the German Siemens 3.0 T Skyra magnetic resonance scanner (eight-channel, head coil). Children were instructed to sleep during image acquisition, followed by routine MRI sequence scans to exclude intracranial organic lesions. The specific parameters were as follows: T2 weighted imaging (T2WI) axis images: repetition time (TR) = 2230 ms, echo time (TE) = 108 ms, matrix = 256 × 256, field of view (FOV) = 240 mm × 240 mm, and slice thickness = 4 mm. T1 weighted imaging (T1WI) axis images: TR = 800 ms, TE = 15 ms, slice thickness = 4 mm, FOV = 240 mm × 240 mm. T2-fluid attenuated inversion recovery (FLAIR): TR = 8000 ms, TE = 102 ms, slice thickness = 4 mm, matrix = 256 × 256, inversion time = 2369 ms, and FOV = 240 mm × 240 mm. The three-dimensional T1-weighted sagittal images were acquired using magnetization-prepared rapid gradient echo: TR = 2300 ms, TE = 2.33 ms, slice thickness = 1 mm, and FOV = 240 mm × 240 mm, scanning time: 4 min 12 s.

### Image Preprocessing

Data were preprocessed using Computational Anatomy Toolbox-CAT12, a toolbox of Statistical Parameter Mapping 12 (SPM 12) software implemented on MATLAB 2012b. According to the CAT12 software analysis, the total brain volume, the volume of GM, white matter (WM), and cerebrospinal fluid (CSF) for each individual can be obtained. Next, individual GM image volumes should be normalized into standard Montreal Neurological Institute (MNI) space with non-linear deformation parameters.

### Individual-Level Brain Network Construction

Distributional divergence-based methods were successfully applied to the construction of individual morphology network (Kong et al., 2014; Wang et al., 2016). Many researchers have utilized the Kullback–Leibler (KL) divergence to construct the individual network:


(1) $$D_{K\,L}\left(P||Q\right)=\int_{-\infty }^{\infty }p\left(x\right)log\frac{p\,\left(x\right)}{q\,\left(x\right)}dx$$

In this equation, the KL divergence is asymmetrical. P and Q represent a pair of ROIs’ probability density function (PDF) of voxel intensity. In our study, we used JSSE to estimate morphological connections between regions to characterize morphological relationships. Compared with KL-based methods, the JSSE method has two advantages. The benefit of this approach is that the range of Jensen–Shannon (JS) divergence (0–1) makes the judgment of similarity more accurate. The second advantage is that it becomes easier to characterize the connections between ROIs because of symmetrical JS divergence.

The detailed process is described as follows (Zhu et al., 2021): first, after preprocessing, the structural T1 images were segmented into GM, WM, and CSF. Next, we used GM to construct individual morphological networks. In detail, we represented brain nodes with the 90 ROIs (45 for each hemisphere without cerebellum) in automated anatomical labeling (AAL) atlas segmentation to describe individual morphological networks. Global normalization was used in each region of interest (ROI) to construct a regional correlation matrix (90 × 90) for everyone. The intensity of the voxels in every ROI was extracted. Then it was used to estimate the PDF of the corresponding ROI with kernel density estimates. Finally, we obtained the morphological connections that are categorized as JS divergence (Li et al., 2021) based on the following mathematical equations:


$$D_{J\,S}\left(P||Q\right)=\frac{1}{2}\left[D_{K\,L}\left(P||M\right)+D_{K\,L}\left(Q||M\right)\right]$$

$$M=\frac{1}{2}\,\left(P+M\right)$$

where M and D_KL_(⋅|⋅) are the KL-divergence. The adjacency matrix describes a pair of morphological connections. And the corresponding elements in it represented the strength of the morphological connection between regions i and j.

### Graph Metrics Construction

In order to explore the alteration of connection patterns in the brain’s morphological networks in ASD, we analyzed the global and local measurement of morphological brain networks using Graph Theory Network Analysis Toolbox (Wang et al., 2015). Specifically, the global metric includes the clustering coefficient (*C_p_*), characteristic path length (*L_p_*), normalized cluster coefficient (γ), normalized characteristic path length (λ), small world (σ), global efficiency (*E*_*global*_), and local efficiency (*E*_*local*_) (Newman, 2004). Local graph metrics also include degree centrality (DC), nodal efficiency (Ne), betweenness centrality (BC), nodal characteristic path length (N*L_p_*), nodal local efficiency (NLe), and nodal clustering coefficient (N*C_p_*). These indicators’ definition could be found in the research of Wang et al. (2015). Different connection patterns can be characterized by global and node graph metrics, as shown in Table 1.

### Feature Selection and ASD Identification

**FIGURE 1:**
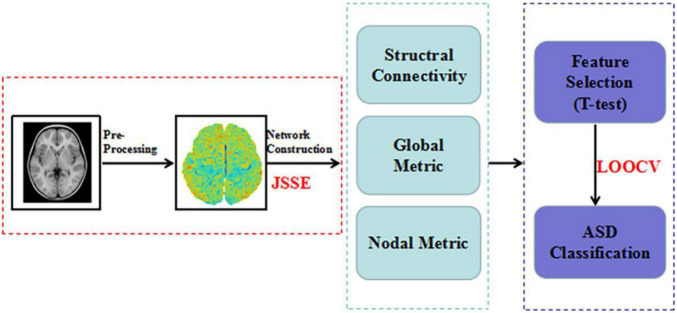
Data processing and analysis.

To confirm the validity of ASD identification, we performed one of the most stringent nest-stay one cross validation (LOOCV) strategies. It can make full use of all subjects, and provides an more accurate classification (Li et al., 2020a). All subjects were used to train classifiers except for one subject. At the same time, to reduce the interference in the feature selection process, we chose Student’s *t*-test (*P* < 0.05) to select the node and global graph measurements (Li et al., 2020b). For connection weights, significance level was set at the 1% level using the Student *t*-test, which was carried out using the non-parametric permutation method (10,000 permutations) (Zuo et al., 2012). Significance levels were set at the 1% level using the Student *t*-test. To combine these information toward better ASD identification, the linear-kernel based MK-SVM is conducted following some recent studies (Xu et al., 2020a,b). Figure 1 provides all procedures mentioned above.

### Statistical Analysis

Statistical analysis was performed using SPSS software (version 25.0, IBM Corporation, Armonk, NY, United States). Continuous variables are expressed as mean ± SD. Student’s *t*-test and Pearson’s χ^2^ test were used for comparisons between two groups. To assess the information combination method and the classification performance of the proposed JSSE, we used the following quantitative measures: accuracy, sensitivity, and specificity. The mathematical definitions of these three measures are given as follows:


$$\text{Accuracy}=\frac{\mathrm{True}\,\mathrm{Positive}+\mathrm{True}\,\mathrm{Negative}}{\mathrm{True}\,\mathrm{Positive}+\mathrm{False}\,\mathrm{Positive}+\mathrm{True}\,\mathrm{Negative}+\mathrm{False}\,\mathrm{Negative}}$$

$$\text{Sensitivity}=\frac{\mathrm{True}\,\mathrm{Positive}}{\mathrm{True}\,\mathrm{Positive}+\mathrm{False}\,\mathrm{Negative}}$$

$$\text{Specificity}=\frac{\mathrm{True}\,\mathrm{Negative}}{\mathrm{True}\,\mathrm{Negative}+\mathrm{False}\,\mathrm{Positive}}$$

The area under the curve (AUC) and the receiver operating characteristic curve (ROC) were calculated as measures for classifying patients with ASD and NCs. Significance levels were set at the 5% level for all, but 1% for morphological connections.

## Results

### Demographics and Clinical Data

Table 2 shows the summary statistics for all participants. No significant differences were found in sex or age between the ASD and NCs (*P* > 0.05 for all).

**TABLE 2:** Demographic and clinical characteristics in ASD patients and NCs.

Variable	ASD (*n* = 24)	NCs (*n* = 17)	*P*-value
Age (months)	32.29 7.32	34.94 7.86	0.275^b^
Sex (female/male)	6/18	5/12	0.753^a^
Gesell	39.92 23.68	NA	NA
ABC	93.25 58.08	NA	NA
M-CHAT	27.75 11.22	NA	NA
CABS	12.676.26	NA	NA

*Gesell, Gesell Developmental Scales; ABC, Autism Behavior Checklist; M-CHAT, Modified Checklist for Autism in Toddlers; CABS, Clancy Autism Behavior Scale.*

*^a^P-value was obtained by using the Chi-square test.*

*^b^P-value was obtained by using a two-sample t-test.*

### Global Graph Metrics of the Morphological Brain Connectome

The global graph metrics of participants in the ASD and NC groups are shown in Table 3. Statistical analyses revealed that there were no significant differences in any of the global graph metrics between participants in the ASD and NC groups (*P* > 0.05 for all).

**TABLE 3:** Global graph measurement of the morphological brain connectome in NCs and ASD.

Global graph metrics	NCs (mean ± SD)	ASD (mean ± SD)
*Ar*	0.1457 0.03	0.1522 0.02
*Q*	17.4754 1.71	17.9321 1.57
*Hr*	0.0582 0.03	0.0688 0.02
*E*_*glocal*_	0.2265 0.01	0.2275 0.01
*E*_*local*_	0.3661 0.01	0.3693 0.01
*C_p_*	0.3146 0.01	0.3166 0.01
γ	1.0225 0.10	1.0645 0.13
λ	0.5602 0.02	0.5543 0.01
σ	0.7879 0.09	0.8247 0.09
*L_p_*	1.0551 0.06	1.0341 0.05
*Sr*	−1.0018 1.25	−1.9170 1.92

*A_r_, assortativity; ASD, autism spectrum disorder; C_p, clustering coefficient; E_global_, global efficiency; E_local_, local efficiency; Hr, hierarchy; L_p, characteristic path length; NCs, normal controls; Q, modularity score; S_r, synchronization; γ, normalized clustering coefficient; λ, normalized characteristic path length; σ, small-world.*

### Nodal Graph Metrics of the Morphological Brain Connectome

The significant differences between the ASD and NCs in each ROI are shown in Tables 4–9. From these tables, it is apparent that the predominant brain regions with different levels of nodal graph measures were distributed mainly in the frontal, occipital, parietal gyri, and basal ganglia (BG). Compared with NCs, patients with ASD had significantly higher values of BC in the IPL.L, MOG.R, and PCL.R (Table 4). For DC, the values of the ASD group were lower than those of the NC group in the HIP.R, LING.R, but higher in IPL.L, ORBmid.R, PCL.L, PUT.L, PUT.R, and THA.R (Table 5). Nevertheless, participants in the ASD group showed significantly lower nodal clustering coefficients in the MOG.R, bilateral ORBmid, and SMA. In PCG.R and PCL.L, the ASD group showed significantly higher values of NLe (Tables 6, 7). For Ne, the ASD group had significantly higher values in IPL.L, ORBmid.R, PCL.L, PCL.R, PUT.L, PUT.R, THA.R compared with the NC group (Table 8) but lower in the HIP.R, LING.R. Nevertheless, in SOG.R, the ASD group showed significantly higher values of N*L_p_*, while lower in ITG.L, MFG.R, SMG.L (Table 9) (*P* < 0.05, for all).

**TABLE 4:** Between-group comparison in BC.

Region	Nodal graph measure	Mean value		*P*-value
		
		NCs	ASD	
MOG.R	BC	20.34094	34.59861	0.037526
IPL.L	BC	49.02761	83.13141	0.012876
PCL.R	BC	8.565475	29.38573	0.015691

**TABLE 5:** Between-group comparison in DC.

Region	Nodal graph measure	Mean value		*P*-value
		
		NCs	ASD	
ORBmid.R	DC	10.444710	12.631250	0.019290
HIP.R	DC	12.156180	8.671000	0.013924
LING.R	DC	14.650590	11.494000	0.032529
IPL.L	DC	10.236760	14.680750	0.013051
PCL.L	DC	3.642941	5.898500	0.039057
PUT.L	DC	8.951765	11.893500	0.014066
PUT.R	DC	8.659706	11.134250	0.040263
THA.R	DC	6.490000	10.792500	0.000206

**TABLE 6:** Between-group comparison in N*C_p_*.

Region	Nodal graph measure	Mean value		*P*-value
		
		NCs	ASD	
ORBmid.L	N*C_p_*	0.342462	0.320918	0.015702
ORBmid.R	N*C_p_*	0.342355	0.321900	0.049634
SMA.L	N*C_p_*	0.370547	0.341055	0.024054
PCG.R	N*C_p_*	0.191760	0.281514	0.034550
MOG.R	N*C_p_*	0.325027	0.294525	0.027890
SMG.L	N*C_p_*	0.315014	0.274908	0.044608
PCL.L	N*C_p_*	0.241941	0.363027	0.006307

**TABLE 7:** Between-group comparison in NLe.

Region	Nodal graph measure	Mean value		*P*-value
		
		NCs	ASD	
ORBmid.L	NLe	0.394183	0.383576	0.042599
SMA.L	NLe	0.406270	0.390297	0.034042
PCG.R	NLe	0.194410	0.284032	0.037018
PCL.L	NLe	0.263333	0.396279	0.003965
THA.R	NLe	0.336583	0.365775	0.040534

**TABLE 8:** Between-group comparison in Ne.

Region	Nodal graph measure	Mean values		*P*-value
		
		NCs	ASD	
ORBmid.R	Ne	0.237425	0.258050	0.009147
HIP.R	Ne	0.249732	0.219698	0.013432
LING.R	Ne	0.271090	0.235913	0.047874
IPL.L	Ne	0.224450	0.271096	0.016760
PCL.L	Ne	0.141197	0.191237	0.019205
PCL.R	Ne	0.153426	0.205746	0.037053
PUT.L	Ne	0.227609	0.254986	0.008673
PUT.R	Ne	0.224428	0.248695	0.024248
THA.R	Ne	0.202564	0.243955	0.000197

**TABLE 9:** Between-group comparison in N*L_p_*.

Region	Nodal graph measure	Mean value		*P*-value
		
		NCs	ASD	
MFG.R	N*L_p_*	3.300256	1.176965	0.048548
SOG.R	N*L_p_*	0.858391	1.471262	0.015142
SMG.L	N*L_p_*	1.459081	0.860388	0.018943
ITG.L	N*L_p_*	3.970017	1.084850	0.039177

### Consensus Significant Morphological Connections

**FIGURE 2:**
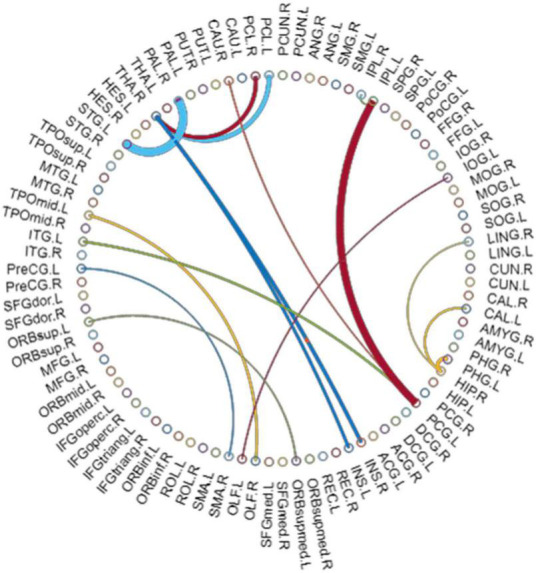
The most consensus connections. The arc thickness indicates the discriminative power of an edge, which is inversely proportional to the estimated *P*-values.

By using Student’s *t*-tests, we selected the consensus connections with *P*-values < 0.01 in each loop, resulting in a total of 16 connections, as shown in Figure 2. We observed that most consensus connections in the frontal, parietal, and thalamic regions were increased inpatients with ASD but decreased in the occipital, prefrontal, and temporal lobes and pallidum. There were 24 nodes with consensus connections, which are listed in Table 10.

**TABLE 10:** Between-group comparison in consensus connections.

Region	Region	Mean value		*P*-value
		
		ASD	NCs	
PAL.R	STG.L	−0.046500	0.071894	0.000197
PCG.L	IPL.L	−0.353380	−1.033010	0.000214
PCL.L	THA.R	−0.629460	−1.055520	0.000397
PCL.R	THA.R	−1.169130	−0.978770	0.000451
INS.R	THA.R	0.389428	−0.149830	0.000523
HIP.R	PHG.L	−1.262140	0.485249	0.000606
INS.L	THA.R	0.358809	−0.304150	0.000690
OLF.R	TPOmid.L	0.326674	0.281932	0.000970
PCG.L	ITG.L	−0.675520	−1.288400	0.001128
HIP.R	CAL.L	−0.987930	0.346887	0.001265
PreCG.L	SMA.R	0.185599	−0.114520	0.001612
SMA.R	PreCG.L	0.185599	−0.114520	0.001612
HIP.R	LING.R	1.253025	0.126837	0.001616
OLF.L	IOG.L	−0.461760	0.310021	0.001773
ORBsup.L	ORBsupmed.L	−1.291030	0.580881	0.001840
IPL.L	IPL.R	0.198135	−0.239100	0.002110

### Classification Results

**FIGURE 3:**
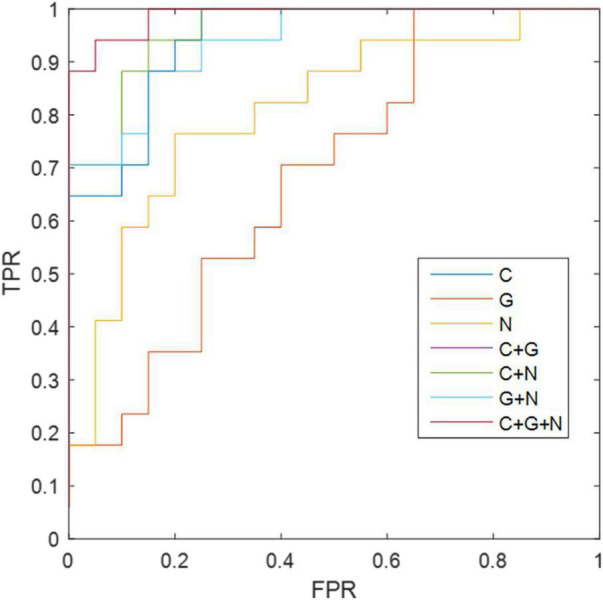
The ROC results of different methods. C, morphological connectivity; G, global metric; N, nodal metric; TPR, true positive rate; FPR, false positive rate.

For the morphological connectivity (C), global metric (G), and node metric (N) of brain network, the corresponding AUC values were 0.9112, 0.6852, and 0.8088 AUC, respectively (Table 11, Figure 3). By combining C and G, G and N, and C and N, we obtained 86.48, 89.20, and 81.08% accuracy, respectively. Interestingly, although the classification ability of global graph metrics is low, it still improve the ability of node graph metrics and morphological connections. Finally, the combination of morphological connection, global metrics, and node metrics (C + G + N) achieves the best classification performance, with an accuracy of 94.59%, a specificity of 95.00%, and an AUC of 0.9882.

**TABLE 11:** Classification performance corresponding to different methods.

Method	Sensitivity (%)	Specificity (%)	Accuracy (%)	AUC
C	82.35	84.00	83.78	0.9112
G	52.94	65.00	59.46	0.6852
N	70.58	80.00	75.67	0.8088
C + G	86.49	85.00	86.48	0.9402
C + N	88.24	90.00	89.20	0.9588
G + N	76.47	86.00	81.08	0.9382
C + G + N	94.11	95.00	94.59	0.9882

*Morphological connectivity (C), global metric (G) nodal metric (N).*

*C + G + N methods are significantly superior to connection, global, and nodal.*

## Discussion

In this study, we selected characterized features from different properties of brain connective groups and combined these information to train the classifier to distinguish between patients with ASD and NCs. Our detailed results are as follows. First, the individual brain network built based on the JSSE method provides multidimensional indicators for individual analysis. Second, patients with ASD affected abnormal brain regions, and their pathways were predominantly distributed in the BG, thalamus, and cortical regions spanning the frontal, temporal, and parietal lobes. The over connection of the above brain regions provides effective brain network features for identifying preschool children with ASD. Finally, the combination of morphological connectivity, global metrics, and node metrics (C + G + N) effectively improves classification performance, and consensus connectivity contributes the most to classification.

Compared with those of participants in the control group, the brain regions with local nodal graph measurements and consensus connections in patients with ASD, differences were mainly distributed in the bilateral precentral gyrus, left inferior parietal, supramarginal and angular gyri, left inferior temporal gyrus, right hippocampus, right lingual gyrus, right thalamus, and right posterior cingulate gyrus. This suggests that the patients with ASD affected abnormal brain regions and that their pathways are predominantly distributed in the BG, thalamus, and cortical regions spanning the frontal, temporal, and parietal lobes, which is consistent with previous studies (Courchesne, 2002; Belmonte et al., 2004; Just et al., 2004; Kumar et al., 2010; Abbott et al., 2018). These brain regions play an important role in social interaction, communication, and repetitive behavior. Although structural abnormalities are not the only mechanism that leads to changes in functional connections, abnormal brain structure, and connections in patients with ASD are one of the theological bases for their abnormal brain function connection patterns (Zikopoulos and Barbas, 2013). The posterior cingulate gyrus is the core hub of the default mode network (DMN) (De Pasquale et al., 2018; Busler et al., 2019) and exhibits the strongest connectivity in its trajectory, especially within the DMN (Gao et al., 2009). The left inferior parietal connects the patterns of action and social cognition and is the key node in the action observation network (AON) (Wymbs et al., 2021). AON is hypothesized to support imitation behavior. When the left inferior parietal is damaged, it may lead to impairment of the core social and communicative characteristics of ASD (Oberman and Ramachandran, 2007). In addition, the thalamus is involved in the processing of neuronal signaling among different cortical regions and is related to cognitive processing and emotion processing. The atypical sensory reactivity seen in ASD could be related to altered thalamic connectivity. ASD-related studies also showed that the thalamus may play a role in sensory overresponsivity (SOR) (Ben-Sasson and Podoly, 2017; Podoly and Ben-Sasson, 2020), an extreme negative response to sensory stimuli (Green et al., 2017).

At the local brain network level, compared with NCs, patients with ASD have a higher value of Ne in the frontal parietal lobe (ORBmid.R, IPL.L, PCL.L, PCL.R), BG (PUT.L, PUT.R), and thalamus, while the limbic system (HIP.R, LING.R) is reduced. In addition, the value of DC in HIP.R and LING.R were decreased. This indicates high input of cortical and BG information, while limbic system information integration and processing efficiency were reduced. The primitive limbic system dominates the control system, which can cause it to be unable to properly regulate external stimuli, thus affecting the child’s ability to think and act. This may be the cause of repetitive stereotyped behaviors and communication disorders in patients with ASD. The increased BC value in patients with ASD in MOG.R, IPL.L, PCL.R regions indicates an enhanced role in the entire brain information transmission system. This study found that the N*L_p_* in the MPG.R, ITG.L of patients with ASD is shorter than that of NCs, indicating that the ability of corresponding brain region function integration is enhanced, and the ability to transmit information over long distances is stronger. Some studies have also reached similar conclusions using diffuse tensor imaging. This abnormality may be related to the WM over connection of the brain of patients with ASD, especially in the network involving the BG and the collateral-limbic system. Moreover, the nodal clustering coefficiency of ORBmid.L, ORBmid.R, SMA.L, MOG.R, SMG.L were also reduced compared to those in NCs, suggesting that the degree of connectivity between those brain regions in the ASD group was reduced, which may be the cause of communication disorders in ASD. However, at the whole-brain level, the means of assortativity, modularity score, hierarchy (*Hr*), *E*_*global*_, *E*_*local*_, clustering coefficient, characteristic path length, and small world in the ASD group were higher than those of NCs but lower in normalized clustering coefficient and normalized characteristic path length. Additionally, there was no significant difference in the comparison between groups, which is the same as the study of Chen et al. (2021). This is different from the result of Gao et al. (2020), which may be related to the tool of morphological connectivity construction.

In addition, this study also showed that the marginal-cortical-basal ganglia-thalamus-cortical circuits in patients with ASD were disturbed. In our analysis of consensus significant morphological connections, the most involved was the cortico-BG-thalamic pathway (Kim et al., 2016). The BG play a crucial role in stereotyped behavior. These structures include the neostriatum (caudal and shell nuclei), globus pallidus, and thalamus and are functionally interconnected. The corticostriatal pathway receives information input from multiple brain regions, and each loop route consists of two distinct pathways: the “direct pathway” (cerebral cortex-striatum-pallidum medial/subthalamic-cerebral cortex) and the indirect pathway (cerebral cortex-striatum-lateral part of the globus-pallidus-subthalamus nucleus-medial palette/subthalamus nigra/subthalamus-cerebral cortex). The BG are involved in regulation through direct and indirect pathways. Any imbalance in these loops can lead to stereotypical behavior. This finding indicates that children with ASD showed overconnectivity within whole-brain networks and internetwork reduction compared to NCs. On the other hand, cortico-subcortical over connection provides a theoretical framework for the existence of social disorders in conceptual autism (Nair et al., 2020). In addition to these regions, our results showed that more connections (16 significant connections in total) of patients with ASD were affected, worthy of further study on a larger scale combined with clinical data.

Our current brain connective approach can effectively distinguish individuals with ASD from HCs because it can measure local network properties and the whole network. In our work, we observed that morphological consensus connectivity and nodal metrics can provide effective indicators for identifying ASD. Although the classification effect of global indicators is the worst, they can still provide information about morphological connections and nodal indicators. By combining morphological connection and nodal metric (C + N), global metric and nodal metrics (G + N), and morphological connection and global metrics (C + G), the classification performance was effectively improved. All of this information is combined to achieve more accurate classification results.

The study has several limitations. First, the study didn’t further classify the severity of ASD patients due to small sample size and imbalanced data. Second, the morphological network of ASD patients will change with aging, and we need to track these patients for further study in the future.

## Conclusion

The individual brain network indicator based on the JSSE method is an effective indicator for identifying individual-level brain network abnormalities in patients with ASD. The proposed classification method can contribute to the early clinical diagnosis of ASD.

## Data Availability Statement

The original contributions presented in the study are included in the article/supplementary material, further inquiries can be directed to the corresponding authors.

## Ethics Statement

The studies involving human participants were reviewed and approved by the Studies Institutional Review Board of Hunan Children’s Hospital. Written informed consent to participate in this study was provided by the participants’ legal guardian/next of kin.

## Author Contributions

TY wrote the first draft of the manuscript. KJ and XG commented on previous versions of the manuscript. All authors contributed to the study conception and design, performed the material preparation, data collection, and analysis, and read and approved the final manuscript.

## Conflict of Interest

The authors declare that the research was conducted in the absence of any commercial or financial relationships that could be construed as a potential conflict of interest. The handling editor ZW declared a past co-authorship with the author XG.

## Publisher’s Note

All claims expressed in this article are solely those of the authors and do not necessarily represent those of their affiliated organizations, or those of the publisher, the editors and the reviewers. Any product that may be evaluated in this article, or claim that may be made by its manufacturer, is not guaranteed or endorsed by the publisher.

## Abbreviations

AAL: automated anatomical labeling
ADHD: attention deficit and hyperactivity disorder
*Ar*: assortativity
AUC: area under the curve
BG: basal ganglia
BOLD: blood oxygen level dependent
*C_p_*: clustering coefficient
DC: degree centrality
DTI: diffusion tensor imaging, *E*_*glocal*_, global efficiency
*E*_*local*_: local efficiency
FLAIR: fluid attenuated inversion recovery
*Hr*: hierarchy
JSSE: Jensen–Shannon Divergence Similarity Estimation
KL: Kullback–Leibler
LOOCV: leave-one-outcross-validation
*L*_p_: characteristic path length
MRI: magnetic resonance imaging
N*C_p_*: nodal clustering coefficiency
NCs: normal controls
Ne: nodal efficiency
NLe: nodal local efficiency
N*L*_p_: nodal characteristic path length
γ: normalized clustering coefficient
Q: modularity score
PDF: probability density function
ROC: receiver operating characteristic curve
sMRI: structural magnetic resonance imaging
T1WI: T1 weighted imaging
T2WI: T2 weighted imaging
TR: repetition time
TE: echo time
FOV: field of view
ROI: region of interest
SPM: statistical parametric mapping
λ: normalized characteristic path length
σ: small-world.
